# Deceased vs living donor grafts for pediatric simultaneous liver‐kidney transplantation: A single‐center experience

**DOI:** 10.1002/jcla.23219

**Published:** 2020-01-22

**Authors:** Sergey Gautier, Artem Monakhov, Olga Tsiroulnikova, Mikhail Voskanov, Igor Miloserdov, Timur Dzhanbekov, Sergey Meshcheryakov, Robert Latypov, Elena Chekletsova, Olga Malomuzh, Khizri Khizroev, Deniz Dzhiner, Irina Pashkova

**Affiliations:** ^1^ Surgical Department #2 National Medical Research Center of Transplantology and Artificial Organs named after V.I. Shumakov Moscow Russia; ^2^ I.M. Sechenov First Moscow State Medical University (Sechenov University) Moscow Russia; ^3^ Surgical Department #1 National Medical Research Center of Transplantology and Artificial Organs named after V.I. Shumakov Moscow Russia; ^4^ Department of Pediatrics National Medical Research Center of Transplantology and Artificial Organs named after V.I. Shumakov Moscow Russia

**Keywords:** autosomal recessive polycystic kidney disease/congenital hepatic fibrosis, living donor, long‐term outcomes, simultaneous liver‐kidney transplantation, split‐liver transplantation

## Abstract

**Introduction:**

In conditions of limited experience of pediatric simultaneous liver‐kidney transplantation (SLKT) using grafts from living and deceased donors, there is a certain need to validate the approach.

**Patients:**

The retrospective study of 18 pediatric patients who received SLKT between 2008 and 2019.

**Results:**

Grafts were obtained from both living and deceased donors. The patients’ age ranged from 2 to 16 years (9 years ±4). The body weight of the children varied from 9.5 to 39 kg (22 kg ±9). The follow‐up period lasted from 1 to 109 months (median 38 months ±35). The various graft combinations were used in both groups. There was no mortality during the follow‐up. There was no significant difference in baseline parameters in recipients who received grafts from living and deceased donors except age (7.5 years ±2.2 vs 11.8 years ±4.1; *P* = .038). Rate of complications > grade II was higher among recipients of deceased donor SLKT (7.7% vs 60%; OR, 7.8; 95% CI, 1.04‐58.48; *P* = .044). All the patients are alive with both grafts functioning. All the living donors returned to the normal life.

**Conclusion:**

SLKT is a safe and effective procedure for children with both simultaneous end‐stage liver disease and end‐stage renal disease. Both living donor partial liver and kidney transplantation and deceased donor liver‐kidney transplantation can be considered as safe and feasible options.

**Abbreviations**
aHUS: atypical hemolytic‐uremic syndrome
ARPKD: autosomal recessive polycystic kidney disease
BMI: body mass index
CHF: congenital hepatic fibrosis
CKD 5: chronic kidney disease, stage 5
CVVH: continuous veno‐venous hemofiltration
GFR: glomerular filtration rate
HD: hemodialysis
ICU: intensive care unit
IVC: inferior vena cava
LL: left lobe
LLS: left lateral section
MELD: model for end‐stage liver disease
MR: magnetic resonance
MV: mechanical ventilation
PD: peritoneal dialysis
PELD: pediatric end‐stage liver disease
PH1: primary type 1 hyperoxaluria
RL: right lobe
RRT: renal replacement therapy
SLKT: simultaneous liver‐kidney transplantation

## 1 INTRODUCTION

The first successful simultaneous liver and kidney transplantation (SLKT) was performed at the University of Innsbruck in 1983 by R. Margreiter. Since that time, SLKT has been established as the treatment of choice for the pediatric and adult patients with simultaneous end‐stage liver and end‐stage kidney disease.^1^, ^2^ In standard clinical practice, the MELD (model for end‐stage liver disease) scoring system is used as a disease severity index to help prioritize the allocation of organs for transplantation. Currently, more than 400 SLKT are performed annually in Europe and the United States. Most of the patients are adults.^2^ In contrast, SLKT is still an extremely rare procedure in children and adolescents. Only approximately thirty pediatric SLKT procedures are performed every year worldwide, and SLKT accounts for 1%‐2% of all pediatric liver transplants. Approximately 1/3 of the recipients are under 5 years of age, and 2/3 of the recipients are between 6 and 17 years old.^3^ Both simultaneous and sequential combined liver‐kidney transplants from the same living donor have been described.^4^ The first successful combined kidney and liver transplantation from a living donor was reported by Marujo et al in 1999.^5^ The first case of laparoscopic partial liver and kidney procurement was described by our transplant team recently.^6^

Combined liver and kidney transplantation can be indicated in pediatric patients for one of the following several reasons: (a) A patient has a disease leading to irreversible hepatic and renal failure, such as an autosomal recessive polycystic kidney disease (ARPKD) with associated congenital hepatic fibrosis; (b) a patient has end‐stage renal failure caused by impaired substance metabolism in the liver, such as in primary type 1 hyperoxaluria (PH1) or atypical hemolytic‐uremic syndrome (aHUS) with mutation of complement factor H^7^ (liver transplantation is performed to correct the underlying defect and prevent disease recurrence in the renal graft^8^); or (c) a patient has acute combined liver and kidney injury (such as drug toxicity or vascular damage). Of note, this final indication is much more commonly observed in adults rather than in children.^9^

For the particular case of children with ARPKD, the indications for liver transplantation include the following: liver failure (with portal hypertension or without), recurrent cholangitis, cirrhosis (verified by biopsy), or “acute” mutations of polycystic kidney disease.^9^, ^10^

The ethical aspect of related organ donation remains controversial, especially in the case of multiorgan living donation.^11^ In pediatric practice, one of the parents of the recipient usually volunteers to become a living donor. Thus, the close emotional relationship normally observed between parents and children can make ethical issues easier to solve.^12^

While several groups have published their limited experiences in pediatric SLKT, more publications are certainly needed to validate the approach across multiple centers.

In the present series, we provide and discuss the results of 18 pediatric patients that underwent combined liver and kidney transplantation at our center.

## 2 PATIENTS AND METHODS

A retrospective review and analysis was performed on prospectively collected data from an institutional database of surgeries, which occurred between March 2008 and May 2019. The median follow‐up period was 38 months ± 35 (1‐109 months). The current study has been approved by the ethics committee of the National Medical Research Center of Transplantation and Artificial Organs, named after academician VI Shumakov.

### 2.1 Patients

During the study period, 816 liver transplants were performed at our center, and 18 of them were combined liver and kidney transplants (in eight boys and 10 girls).

When a patient with ARPKD with CHF is listed for CLKT, the following principles are taken into account: liver function (PELD score), presence of portal hypertension, quality of life (skin itching), and repeating severe biliary infections.

In 13 cases, the liver and kidney transplants were procured from living donors, and in five cases, they were procured from deceased donors. The first pediatric SLKT using the grafts from a deceased donor in our center was performed in August 2017. In two cases, liver and kidney grafts were procured from the same living donor using a purely laparoscopic approach.

Hepatic grafts were represented by (a) the right hepatic lobe in six cases (five grafts from living donors and one split graft from a deceased donor), (b) the left lobe in four cases (all from living donors), (c) the left lateral section in six cases (four grafts from living donors and two split grafts from deceased donors), and (d) the whole liver in two cases.

Complications were estimated according to Clavien‐Dindo Classification.^13^, ^14^ Postoperative biliary leakage identification and grading were based on ISGLS classification.^15^

### 2.2 Selection of the living donors

According to Russian law, only genetic relatives may be considered as living donors. Primary assessment of all donors included estimation of general health and obtaining informed consent. Next, a potential donor underwent laboratory, instrumental, and functional examination. Finally, liver and kidney anatomy and function assessments were carried out. In particular, preoperative evaluation included the analysis of the vascular anatomy (via CT scan), the biliary anatomy (via MR cholangiography), and the tissue suitability (via liver biopsy). Furthermore, the inclusion criteria for the laparoscopic procedure additionally included the presence of standard arterial (Michaelis types I‐III) and venous anatomy.

### 2.3 Split‐liver procedure (deceased donors)

Donors with brain death younger than 45 y.o. without limitations (hemodynamically stable with the use of ≤2 vasopressors, <5 days in ICU, serum sodium level <160 mmol/L, liver ultrasound without evidence of steatosis, normal or subnormal total serum bilirubin and transaminases) were considered for split‐liver transplantation. The final decision was made after gross liver examination as well as arterial anatomy evaluation. In situ liver resection was applied as a method of choice in order to reduce the graft cold ischemia time. Conversion to ex situ splitting on the back bench was performed if hemodynamical instability developed during the resection.

### 2.4 Surgical technique

At least a day before the surgery, all the patients undergoing renal replacement therapy (RRT) had been given a hemodialysis session.

The surgical technique of SLKT does not significantly differ from an isolated liver or kidney transplantation. Depending on the anthropometric characteristics of the recipient, the following graft combinations were applied: left lateral section and kidney, left lobe and kidney, right lobe and kidney, or whole liver and kidney.

An appropriate graft type was chosen based on the weight of the patient, the abdominal cavity size of the recipient, the availability of the organ for transplantation, and the severity of the underlying disease.

All procedures were done using our institution's stepwise SLKT surgical approach included consequential steps: vascular reconstruction of the liver graft (caval anastomosis, portal anastomosis, followed by reperfusion and arterial anastomosis), vascular reconstruction of the kidney graft (followed by kidney reperfusion and ureterocystoanastomosis), and biliary reconstruction. This particular series of steps reduces both hepatic warm ischemia and renal cold ischemia to the minimal time necessary.

Bilateral nephrectomy was performed in all of the recipients in order to prevent infection and malignancy of the native kidneys in the future.

Ureterocystoanastomosis was accompanied by routine stent placement. The biliary reconstruction was performed as a Roux‐en‐Y choledochojejunostomy in 14 cases (77.8%) and as duct‐to‐duct anastomosis in four cases (22.2%). Stent drainage was applied occasionally based on the diameter of the duct.

### 2.5 Immunosuppression

Induction of immunosuppression included basiliximab. Methylprednisolone (5 mg/kg) was administrated twice during the surgery: first after liver reperfusion and again after kidney reperfusion. The basic immunosuppressive protocol included tacrolimus, low‐dose methylprednisolone, and mycophenolate mofetil (MMF). During the first 3 months after SLKT, the concentration of tacrolimus in the blood was maintained at the level of 7‐12 ng/mL. Individual side effects of MMF such as diarrhea or leukopenia were considered outcomes, which triggered consideration for discontinuation of the drug.

### 2.6 Statistical analysis

Continuous variables were reported as mean ± standard deviation (SD) and compared using the Student *t* test or the Mann‐Whitney *U* test. Fisher's exact test was used for categorical variables. Odds ratio (OR) was expressed with 95% confidence interval (CI) and p‐value. Differences with *P*‐value < .05 were considered as statistically significant. Statistical analysis was performed via SPSS software (version 23.0).

## 3 RESULTS

The baseline perioperative parameters of the recipients such as age, gender, body weight, diagnosis, presence of renal replacement therapy before the transplantation, graft type, RRT before surgery, ABO compatibility with the donor, graft‐to‐recipient weight ratio, recipient surgery duration, and immunosuppressive regimen are summarized in Table 1. Baseline parameters of the donors such as age, sex, BMI, and relation to the recipient are reflected in Table 2.

**Table 1:** Perioperative parameters of the recipients

Variables	SLKT from living donor n = 13	SLKT from deceased donor n = 5	*P*‐value
Age, median (±SD), y	7.5 (±3.6)	11.8 (±3.3)	**.038**
Weight, median (±SD), kg	19.9 (±7.5)	28.4 (±10.5)	.073
Sex, n (%)			
Male	6 (46.2)	2 (40)	.827
Female	7 (53.8)	3 (60)	.827
RRT before surgery, n (%)			
Hemodialysis	4 (30.8)	1 (20)	.648
PD	3 (23.1)		.239
Combined hemodialysis and PD	1 (7.7)	4 (80)	**.002**
None	5 (38.5)	‐	.103
Indication, n (%)			
ARPKD/CHF	12 (92.3)	5 (100)	.535
Alagille/renal hypoplasia	1 (7.7)	‐	.535
Liver graft type, n (%)			
LLS	4 (30.8)	2 (40)	.710
LL	4 (30.8)	1 (20)	.160
RL	5 (38.4)	2 (40)	.457
Whole liver	‐	‐	‐
ABO compatibility, n (%)			
Compatible	11 (84.6)	5 (100)	.366
Incompatible	2 (15.4)	‐	.366
GRWR, median (±SD), %	2.7 (± 0.3)	3 (±1.3)	.359
Operation time, h (±SD)	8.7 (± 1.9)	10.5 (±1.1)	.083
Primary kidney graft function, n (%)	13 (100)	4 (80)	.074
Immunosuppressive regimen, n (%)			
Tac + MP	4 (30.8)	1 (20)	.671
Tac + MMF	1 (7.7)	‐	.551
Tac + MP +MMF	8 (61.5)	4 (80)	.486
Complications (Clavien‐Dindo), n (%)			
I	1 (7.7)	‐	.551
II	1 (7.7)	‐	.551
IIIa	1 (7.7)	2 (40)	.099
IIIb	‐	1 (20)	.097
IV	‐	‐	‐
V	‐	‐	‐
Follow‐up, median (± SD), mo	48.3 (±36.6)	11.2 (±6.5)	**.042**
Overall mortality, n (%)	0	0	**‐**

Statistically significant *P*‐values (*P* < .05) are indicated in bold font.

Abbreviations: ARPKD, autosomal recessive polycystic kidney disease; CHF, congenital hepatic fibrosis; GRWR, graft‐to‐recipient weight ratio; LL, left lobe; LLS, left lateral section; MMF, mycophenolate mofetilMP, methylprednisolone; PD, peritoneal dialysis; RL, right lobe; RRT, renal replacement therapy; SLKT, simultaneous liver‐kidney transplantation; Tac, tacrolimus.

**Table 2:** Baseline characteristics of liver‐kidney living donors

Parameters	Liver‐kidney living donors, n = 13
Age, median (± SD), y	35.5 (±3.5)
Sex, n (%)	
Male	2 (15.4)
Female	11 (84.6)
Relation, n (%)	
Mother	11 (84.6)
Uncle	2 (15.4)
BMI, median (±SD), kg/m^2^	22.7 (±1.5)
Open, n (%)/ lap, n (%)	11(84.6)/2 (15.4)
Complications > grade II (Clavien‐Dindo), n (%)	
IIIa	2 (15.4)
IIIb	1 (7.7)
IV	‐
V	‐

The age of the patients ranged from 2 to 16 years (9 years ±4). The weight varied from 10 to 38 kg (22.5 kg ±9.5). The indications for transplantation included autosomal recessive polycystic kidney disease (ARPKD) in combination with congenital hepatic fibrosis (CHF) in 17 cases, as well as Alagille syndrome in combination with bilateral renal hypoplasia in one case. All the patients had an advanced chronic kidney disease with glomerular filtration rate of less than 30 mL/min.

Thirteen patients were undergoing various RRT prior to surgery: hemodialysis (HD; n = 5), peritoneal dialysis (PD; n = 3), and combined HD + PD (n = 5). Five patients did not require RRT before the transplantation. All of these recipients have obtained grafts from living donors, but there was no significant difference between the groups (*P* = .11).

In all cases, the kidney and liver transplants were procured from the same donor. In three cases of split‐liver grafts, two were LLS grafts after the “classical” split and one was a right liver graft obtained by “full left/full right” splitting.

No mortality occurred among the living liver donors. In 11 cases, the related donors were the mothers of the patients and in two cases the uncles. The age of the donors ranged from 25 to 47 years (35.5 ± 3.5), BMI from 20 to 26 kg/m^2^ (22.7 ± 1.5).

After the surgery, all the patients were observed in the intensive care unit (ICU) while requiring mechanical ventilation (MV). The average time spent by recipient on MV in the ICU was 9 (±7.7) hours. One patient required CVVH in the ICU due to delayed renal graft function, which resolved after the appropriate treatment. Other than age and follow‐up (Table 1), there were no statistically significant relationships found between the perioperative parameters of the recipients and their respective donation source (living vs deceased).

### 3.1 Morbidity

Complications occurred in 6 (33.3%) recipients. Prolonged lymphorrhea occurred in 2 (17.6%) patients: In 1 (5.5%) case, complication required prolonged standing drainage (<1 week). And in one case (5.5%) lymphocele formed, which also required prolonged drainage (more than 1 week). Wound eventration occurred in one patient (5.5%), which has been successfully managed by vacuum‐assisted closure wound therapy. Also, 1 (5.5%) patient developed hepatic artery steal syndrome, which was resolved by selective embolization of the splenic artery. During the time of the study, the readmission rate was 5.5% (n = 1) due to liver graft dysfunction caused by biliary stricture. Besides, four patients have been at least once admitted to a local hospital due to viral infection (n = 1; 5.5%), MMF‐induced leukopenia (n = 1; 5.5%), seizures (n = 1; 5.5%), and motor vehicle collision (n = 1; 5.5%) with no long‐term negative consequences. The overall rate of complications greater than Clavien‐Dindo grade II was significantly higher in the group of deceased donor SLKT (OR, 7.8; 95% CI, 1.04‐58.48; *P* = .04).

**Figure 1:**
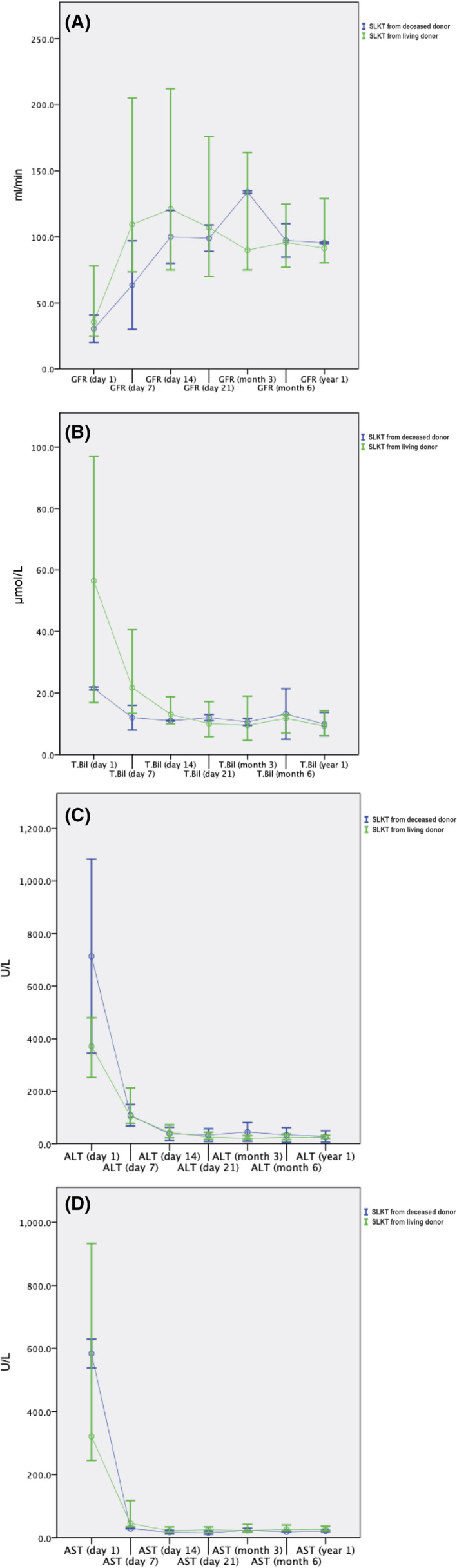
Postoperative course after SLKT. Dynamics of laboratory parameters in recipients of grafts from deceased (blue) and living donors (green): (A) glomerular filtration rate (GFR), Schwartz formula; (B) level of total bilirubin (T.Bil); (C) alanine aminotransferase (ALT); and (D) aspartate aminotransferase (AST)

Complications greater than Clavien II have been found in three living donors (23.1%). Two of these cases were biliary leakage (ISGLS grade B), which were successfully resolved by placement of a percutaneous drain. In one case, a donor required a second operation to correct a non‐resolving (>4 weeks) biliary fistula. All of the patients are alive and have achieved satisfactory function of both grafts (Figure 1). All the related donors returned to their normal professional and recreational activities. No donor displayed evidence of abnormal liver or kidney functioning during the entire observation period.

## 4 DISCUSSION

Few recent studies have been published on this subject.^16^, ^17^, ^18^ All of the papers, including this work, provide a very limited series of combined liver‐kidney transplantations in children (from 4 to 40 cases). This study is notable for the broad use of a living donor pool and deceased donor grafts. Long‐ and short‐term results in both recipients and living donor were provided. There was a limited attempt to compare the approaches. Mortality, graft failure, or their function during 1‐year follow‐up was not different between the groups. However, the composite of Clavien‐Dindo IIIb‐V complications was more frequent in the deceased donor SLKT group.

Excellent recipient and graft survival rates were demonstrated, although this may be related to the selection of stable recipients with ARKPD and congenital hepatic fibrosis. In addition, five of the SLKT procedures were performed on dialysis‐free patients.

A unique feature of this work is also its demonstrated application of pure laparoscopic approach for organ procurement from two living donors (n = 2; LLS + kidney and LL + kidney). The laparoscopic living donor hepatectomy has been rapidly spreading among high‐volume centers for the past few years. Laparoscopic kidney transplantation has already been established as the gold standard for kidney donors. The combination of these two procedures can gain from the traditional benefits of minimal surgery (eg, reduced pain and blood loss, shorter duration of hospital stay, and enhanced rehabilitation) in particular. However, it is a subject for further investigations.

Our study, in agreement with others, confirmed that the most common indication for SLKT is the ARPKD associated with congenital hepatic fibrosis.^18^, ^19^, ^20^, ^21^

Indications for liver transplantation included evaluation of liver function and the presence of portal hypertension. Moreover, children with CHF often have repeating severe cholangitis, accompanied by high intoxication and skin itching, the course of which only becomes malignant during immunosuppressive therapy after isolated kidney transplantation.

Conspicuous is the absence of patients with primary hyperoxaluria type 1. In agreement with other studies,^22^, ^23^ we believe that sequential liver and kidney transplantation is preferable to simultaneous surgery for patients with hyperoxaluria because it allows for the disease‐associated metabolic disorder to correct before kidney transplantation.

We always prefer a single donor for both kidney and partial liver donation. If the child has a relative who expresses a desire and consent to be a donor, we provide him with this opportunity. If the child does not have relatives expressing an active desire to become a donor, then the patient is ordered to the waiting list. We believe that using two different donors for one child is justified only in cases of sequential transplantation, for example, PH1 cases.

Donor morbidity is a paramount topic especially when considering two graft procurements. Three donors with more than Clavien‐Dindo grade II complications have been observed. In the present series, three of 13 donors experienced biliary leakage. All of these complications developed after major open hepatectomy (right‐ or left‐sided). Kidney function stayed sufficient in all the donors during the follow‐up. Therefore, we can assume that liver complications prevail over kidney complications after combined procurement. At the same time, the complication rate is comparable to liver‐alone donation. However, more multicenter studies are needed to confirm these hypotheses.

Unlike sequential liver and kidney transplantation, there was no need for programmed hemodialysis after the surgery (in cases of sufficient renal graft function), which spared the patients from heparin infusion in the early postoperative period. As a result, they were also spared the installation of additional vascular ports or arteriovenous fistula formation. Moreover, we did not observe any cases of depression in living donors, when Kitajima et al described cases of depression among donors between surgeries in sequential liver‐kidney transplantation.^4^ This finding makes us think that simultaneous approach may have a positive impact on the incidence of surgery‐related mood disorders in living donors.

In summary, our work shows that SLKT can be successfully applied for ARKPD/CHF patients with promising long‐term outcomes.^24^, ^25^, ^26^, ^27^, ^28^ Both split‐liver‐kidney from deceased donors and living donor liver‐kidney approaches are effective procedures with similar outcomes in the recipients. At the same time, we believe that living donor morbidity can be diminished and rehabilitation can be enhanced with the implementation of a laparoscopic minimally invasive approach.

## 5 CONCLUSION

Pediatric simultaneous liver and kidney transplantation is a safe and effective method of treatment for children with simultaneous end‐stage liver failure and end‐stage renal failure. Living donors can be considered for simultaneous partial liver and kidney procurement. In addition, deceased donors can remain an important source of grafts for combined pediatric liver‐kidney transplantation since both split‐liver‐kidney transplants from deceased donors and living donor liver‐kidney transplants have shown similar outcomes in the recipients.

## CONFLICT OF INTEREST

The authors declare no conflicts of interest.
